# Feeling the heat: Long-term heat stress impairs growth but not photosynthesis in a C_4_ grass

**DOI:** 10.1093/plcell/koaf008

**Published:** 2025-01-07

**Authors:** Guy Levin, Gadi Schuster

**Affiliations:** Assistant Features Editor, The Plant Cell, American Society of Plant Biologists; Faculty of Biology, Technion, Haifa, 32000, Israel; Faculty of Biology, Technion, Haifa, 32000, Israel; Grand Technion Energy Program, Technion, Haifa, 32000, Israel

Long-term heat stress negatively affects plants in multiple ways, including reducing the stability of photosynthetic and other proteins and enhancing photorespiration. In photorespiration, the carbon-fixing enzyme Rubisco uses O_2_ as a substrate instead of CO_2_, thus suppressing carbon fixation (Bauwe et al. 2010). Some plants that evolved under photorespiration-favoring conditions, such as high temperatures, developed C_4_ photosynthetic metabolism that reduces photorespiration. In C_4_ metabolism, CO_2_ is actively concentrated near Rubisco, increasing the likelihood of Rubisco interacting with CO_2_ instead of O_2_ (Sage 2004). However, despite their high photosynthetic efficiency under heat stress, the effects of extreme weather events such as heat waves on C_4_ plants are unclear.

In this issue, **Peng Zhang and colleagues Zhang et al. (2025)** use a systems approach to analyze long-term heat stress responses in the C_4_ grass *Setaria viridis* to determine their effects on photosynthesis and growth. To replicate heat waves, *S. viridis* was grown for 2 weeks at a 42 °C/32 °C day/night cycle compared with a 28 °C/22 °C day/night cycle for control plants. Heat-stressed plants displayed stunted growth (Figure) and anatomical changes, including an approximately 50% reduction in plant biomass and smaller leaves. Contrarily, photosynthesis was less affected, as indicated by similar maximal photosynthetic rates and Rubisco activity in the heat-stressed plants relative to control plants. Moreover, the responses of the photosynthetic rate to varying CO_2_ concentrations or light intensities were similar between the treatments (Figure) and so was the response of carbon assimilation to changing temperatures. Nevertheless, the optimal temperature for photosynthesis increased in heat-stressed plants, suggesting they at least partially adapted to the heat. Together, these results point to a strong homeostatic response where photosynthesis retains maximal activity even while growth is stunted, reducing heat-induced vegetative damage to the plants.

**Figure.**
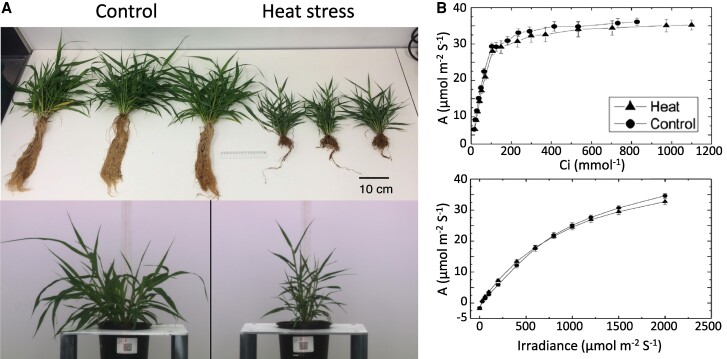
Long-term heat stress impairs growth but not photosynthesis in *Setaria viridis*. **A)** Plants grown at 28 °C day/22 °C night (control) and 42 °C day/32 °C night (heat stress) cycles. **B)** The CO2 assimilation rate (*y*-axis) of the flag leaf in response to increasing concentrations of CO_2_ (top) or light intensity (bottom). Adapted from Zhang et al. (2025), Figures 1 and 2.

Further, the authors generated transcriptomic, proteomic, and metabolomic profiles of heat-stressed and control plants. The results showed an increased flux in the pathway leading to phosphoenolpyruvate production from aspartate, suggesting aspartate is an important acid of the C_4_ cycle under long-term heat stress. A Rubisco activase isoform with an extended C terminal was more highly expressed in heat-stressed plants; consistent with a previous report that longer Rubisco activase isoforms are more stable under heat stress (Ristic et al. 2009). Although C_4_ plants perform limited photorespiration relative to C_3_ plants, some proteins involved in the photorespiratory cycle and glycerate, a product of the cycle, were more highly accumulated in *S. viridis* under heat stress compared with nonstress conditions. Sugar and starch content analysis showed that heat-stressed plants accumulated more soluble sugars while starch levels were reduced, suggesting a shift in their metabolism relative to the control plants. Soluble sugar accumulation may promote heat stress tolerance by regulating osmotic stress through maintaining turgor pressure or stabilizing proteins (Lunn et al. 2014). Moreover, the shifts in sugar content may have a profound impact on sugar signaling pathways. Finally, leaf hormone levels were measured to investigate their role in the retarded growth of heat-stressed plants. While most hormone levels remained similar under both growth conditions, abscisic acid, known to inhibit growth in certain concentrations (Kavi Kishor et al. 2022), showed significant accumulation in the heat-stressed plants. Transcriptomic analysis further supported this connection, showing increased expression of genes involved in the abscisic acid pathway.

C_4_ metabolism allows crops like corn and sugarcane to be highly productive under elevated light and temperature growth conditions. With increasing global temperatures, there is interest in using biotechnology tools to engineer C_4_ metabolism into important C_3_ crops, such as rice (Hibberd et al. 2008) to improve photosynthetic efficiency and increase crop yields. Uncovering the molecular processes of C_4_ photosynthesis under long-term heat stress is an important step toward achieving this goal. The work of Zhang et al. (2025) illustrates the complexity of long-term heat stress responses and the need for more whole-system approaches to understand the interplay of growth and photosynthesis under elevated temperatures.

## Data Availability

No data analysis was performed during this work.
